# Three Gorges Dam: polynomial regression modeling of water level and the density of schistosome-transmitting snails *Oncomelania hupensis*

**DOI:** 10.1186/s13071-018-2687-x

**Published:** 2018-03-14

**Authors:** Ya Yang, Jianchuan Gao, Wanting Cheng, Xiang Pan, Yu Yang, Yue Chen, Qingqing Dai, Lan Zhu, Yibiao Zhou, Qingwu Jiang

**Affiliations:** 1 Fudan University School of Public Health, Building 8, 130 Dong’an Road, Xuhui District, Shanghai, 200032 China; 2 Key Laboratory of Public Health Safety, Fudan University, Ministry of Education, Building 8, 130 Dong’an Road, Xuhui District, Shanghai, 200032 China; 3 Fudan University Center for Tropical Disease Research, Building 8, 130 Dong’an Road, Xuhui District, Shanghai, 200032 China; 4 School of Epidemiology and Public Health, Faculty of Medicine, University of Ottawa, 600 Peter Morand Crescent, Ottawa, ON K1G 5Z3 Canada; 5 Department of Statistics, Oklahoma State University, Stillwater, 74078 USA

**Keywords:** *Oncomelania hupensis*, Schistosomiasis, Polynomial regression, Flooding duration, Snail density

## Abstract

**Background:**

Schistosomiasis remains a major public health concern in China. *Oncomelania hupensis* (*O. hupensis*) is the sole intermediate host of *Schistosoma japonicum*, and its change in distribution and density influences the endemic *S. japonicum*. The Three Gorges Dam (TGD) has substantially changed the downstream water levels of the dam. This study investigated the quantitative relationship between flooding duration and the density of the snail population.

**Methods:**

Two bottomlands without any control measures for snails were selected in Yueyang City, Hunan Province. Data for the density of the snail population and water level in both spring and autumn were collected for the period 2009–2015. Polynomial regression analysis was applied to explore the relationship between flooding duration and the density of the snail population.

**Results:**

Data showed a convex relationship between spring snail density and flooding duration of the previous year (adjusted *R*^2^, *aR*^2^ = 0.61). The spring snail density remained low when the flooding duration was fewer than 50 days in the previous year, was the highest when the flooding duration was 123 days, and decreased thereafter. There was a similar convex relationship between autumn snail density and flooding duration of the current year (*aR*^2^ = 0.77). The snail density was low when the flooding duration was fewer than 50 days and was the highest when the flooding duration was 139 days.

**Conclusions:**

There was a convex relationship between flooding duration and the spring or autumn snail density. The snail density was the highest when flooding lasted about four to 5 months.

## Background

Schistosomiasis is a global public health issue that affects more than 230 million people in approximately 76 countries with a loss of 1.53 million disability-adjusted life years (DALYs) [1–3]. It is caused by trematode parasites of the genus *Schistosoma*, of which *Schistosoma japonicum* is the only human blood fluke that occurs in southern China and large parts of the Philippines [3]. During the past six decades, comprehensive control measures have been taken and schistosomiasis has been successfully controlled in most parts of China [4, 5]. The number of people infected with *S. japonicum* decreased from 11.6 million in 12 provinces, municipalities, and autonomous regions in the mid 1950s to 77,194 in nine provinces in 2015 [4, 6]. Approximately 95% of current schistosomiasis cases occur in the lake and marshland regions of Hunan, Hubei, Jiangxi, Anhui and Jiangsu Provinces with vast areas of *O. hupensis* habitats [6], where the control of schistosomiasis transmission is difficult.

The distribution of *S. japonicum* is closely associated with that of *O. hupensis*, which largely depends on environmental conditions such as vegetation coverage, temperature, soil type and water level [7, 8]. Understanding how water level affects *O. hupensis* is crucial to the control of schistosomiasis. Newly hatched *O. hupensis* are aquatic; however, adult snails are amphibious and cannot withstand prolonged submersion [9]. Hence, seasonal flooding decreases the density of the snail population [7]. Water level regulation has been used to control the snail population, which led to significant reductions in snail density, snail infection rates and human-water contact likelihood [5, 10].

It has been reported that the management of water resources is an important influencing factor for the control of schistosomiasis [11]. The Three Gorges Dam (TGD) is a world-class water conservancy project located in the upper reaches of the Yangtze River of which the middle and lower reaches are the largest endemic areas of schistosomiasis in China. Hence, the potential impact of the TGD on *O. hupensis* snails and *S. japonicum* in the Yangtze River basin has received a worldwide attention [12]. The impact of the water level changes on *O. hupensis* snails varied from segment to segment of the Yangtze River. Generally, the construction and operation of TGD has led to higher water levels for the first quarter of a year and lower water levels for the remaining time [12]. Such a change in water levels means earlier occurrence and recession of the annual flood, which would disturb the life-cycle of *O. hupensis* snails. A subsequent reduction in snail density and/or changes in snail distribution has been observed at the downstream areas of TGD, including Lakes Dongting and Poyang [12, 13].

Generalized additive models (GAMs) have been applied to explore the effects of microecological environmental factors, including water level, on the density of *O. hupensis* snail populations [14, 15]. Hong et al. [14] found a quadratic curvilinear relationship between spring snail density and flood duration of the previous year. However, GAMs could not derive a quantitative relationship between the snail density and water level change. In this study, we aimed to determine the quantitative relationship between flooding duration and spring or autumn snail densities using polynomial regression model in order to develop a more effective snail control strategy.

## Methods

### Study area and malacological surveys

This study was conducted in two bottomlands (Junshan: 29°21′19.42″N, 113°0′4.05″E, approximately 48,000 m^2^; Changgouzi: 29°28′48.12″N, 112°58′34.97″E, approximately 768,900 m^2^) in Junshan District, Yueyang, Hunan Province. The areas of both bottomlands are located outside of the embankment and have similar vegetation and micro-environmental characteristics. This study area is classified as the East Asian monsoon climate zone. The average annual precipitation was 1369.1 mm and the annual average temperature was 17.8 °C during 1996–2015. Those grasslands are also typical endemic areas of *S. japonicum* and marshland habitats for the *O. hupensis* snails [5], which underwent no previous snail control interventions.

The two bottomlands are public lands so our snail survey did not require a permit or consent. A routine biannual survey of *O. hupensis* snails was carried out in April (pre-flood) and November (post-flood), from 2009 to 2015. Systematic quadrat sampling was adopted for snail collection in this study [13, 16]. This sampling strategy is now designated as the national standard for *O. hupensis* snail surveys [17] in which setting of the quadrats and the line spacing should be determined according to the size of bottomlands. One hundred and twenty quadrats were set up in Junshan and 1600 in Changhgouzi. A number of parallel survey lines were set up on the ground with an equal distance of 20 m. Along the survey lines, a 0.11 m^2^ frame made of iron wire was placed at every 20 m interval. All snails within the frame were collected and counted. The density of snails was calculated as the average number of snails per frame (Number/ 0.11 m^2^).

### Water level data

Data on the daily water level at 8:00 am from 2009 to 2015 were collected from the Chenglingji hydrological station (29°26′5.60″N, 113°8′54.81″E), located in the East Dongting Lake and the Yangtze River confluence, approximately 15 km from the two study sites. This station reflects water level changes for the two bottomlands. We used the GeoExplorer 2008 GPS receiver (Trimble Navigation Inc., Sunnyvale, USA) to measure the elevation of the study sites. Both bottomlands are flat, with an elevation difference no more than 0.5 m. An average elevation was calculated for each bottomland. The elevations of the bottomlands and Chenglingji hydrological station were measured based on the same reference geoid and sea level at the lowest tide measured at Shanghai Wusongkou Tide Station. The bottomland was considered as flooded if the difference between the daily water level at the hydrological station and the average elevation of the bottomland was greater than zero. Flooding duration was defined as days from the onset of flooding to the offset in a year. Flooding timing for a year was defined as days from January 1 to the beginning of flooding.

### Statistical analysis

Summary statistics including median, minimum, maximum and standard deviation of all variables were calculated. Box-whisker plots were used to show the distributions of each predictor variable and snail density.

Flooding duration is an important determinant of the snail density. *Oncomelania hupensis* hatchlings develop in water and begin to leave the water as early as 3 week-old [18]. However, they must live under water when all the vegetation is submerged during the flood season. As for adult snails, these cannot survive a long duration of flooding so that the snail population in autumn is mainly composed of snails hatched within the year. Flood duration was highly negatively correlated with flooding timing (Pearson’s *r* = -0.77, *P* <  0.01).

Due to a seasonal effect, the snail density was modeled separately for spring and autumn. The distribution of snail densities was checked with Shapiro-Wilk normality test. We used the maximum likelihood-like approach of Box & Cox [19] to select an appropriate transformation of snail densities [20]. Results showed that the log transformation of snail densities was adequate. Log-transformed spring and autumn snail densities were highly positively correlated with each other (Pearson’s *r* = 0.95, *P* <  0.001). Inclusion of log-transformed spring snail density in the autumn density model prevented us from studying the relationship between flood duration and snail density. For this reason, spring density was not included as a co-variate, and this was also the case for the spring snail density model. Polynomial regression was employed to model the relationship between flooding duration and snail density. Flooding duration of the current year (t_1_) was used for the autumn snail density modeling while its previous year’s flooding duration (t_2_) was used for the spring snail density modeling. The initial polynomial regression models for spring and autumn were built by the following equation:

$$ \mathrm{Y}={\beta}_0+{\beta}_1t+{\beta}_2{t}^2+{\beta}_3{t}^3 $$

where Y is natural logarithm transformed snail density, and *β*_*0*_, *β*_*1*_*, β*_*2*_ and *β*_*3*_ are the regression coefficients.

The initial models were reduced by a backward selection approach of removing the term with the highest *P*-value. The final models were determined based on the significance of variables and scientific judgment. For the present study, if the significance level was set at 0.05, the final models would mean that snail density increased monotonically with flood duration. There was no plausible biological explanation for this kind of relationship. We chose models that agreed with field observations. Adjusted *R*^2^ (*aR*^2^) was calculated to evaluate the goodness-of-fit for the final models. We used the Durbin-Watson test to detect the presence of autocorrelation in the residuals [20]. The inflection point corresponding to a peak snail density was identified using the first derivative of the model equation. All statistical analyses were performed in R version 3.3.1 software. Evaluation of model assumptions and Durbin-Watson test were implemented with the *gvlma* package [21] and *car* package [20].

## Results

### Descriptive analysis

**Fig. 1:**
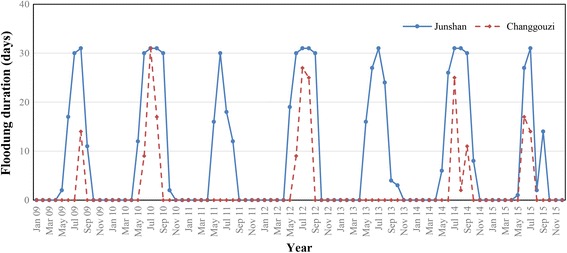
Monthly flooding duration for Junshan and Changgouzi bottomlands from 2009 to 2015

Figure 1 summarizes the monthly variations of flooding duration for the two bottomlands. Junshan had a similar flooding duration each year, starting in May and ending in September. Changgouzi showed a considerable variation in flooding duration over the study years, with an absence of submersion in 2011 and 2013.

**Fig. 2:**
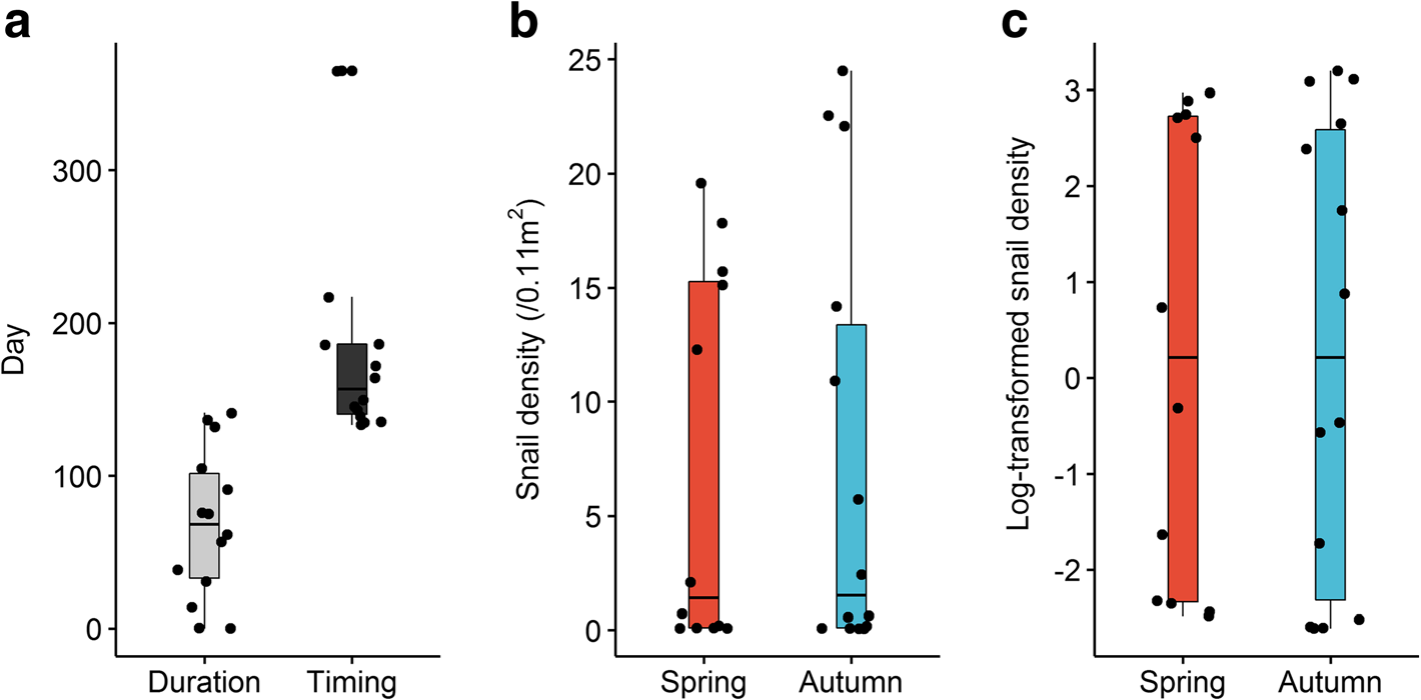
Boxplots showing duration and timing of flooding (**a**), snail density (**b**) and log-transformed snail density (**c**). Boxes encompass 25th to 75th percentiles, horizontal lines within the boxes represent the median, whiskers extend to the most extreme point within 1.5 interquartile ranges (IQRs) of the box and dots outside boxes indicate outliers

The flooding duration ranged from 0 to 141 days during 2009–2015, with an average of 68 days. During the same period, the flooding timing was also highly variable, ranging between 133 and 365 days (Fig. 2a), with an average of 188 days.

The non-transformed and transformed snail densities in the spring and autumn are shown in Fig. 2b, c. For the period 2009–2015, the spring snail density ranged between 0.08–19.58 snails/0.11 m^2^ and the autumn snail density ranged between 0.07–24.50 snails/ 0.11 m^2^. The geometric means of the spring and autumn densities were 1.29 and 1.33 snails/ 0.11 m^2^, respectively Shapiro-Wilk normality tests showed that the snail density was not normally distributed for both the spring (*W* = 0.76, *P* = 0.003) and autumn (*W* = 0.76, *P* = 0.001). The transformed snail densities showed an improvement but still did not reach normality (*W* = 0.80, *P* = 0.010 and *W* = 0.85, *P* = 0.022, respectively).

### Polynomial regression modeling

#### Spring snail density modeling

**Fig. 3:**
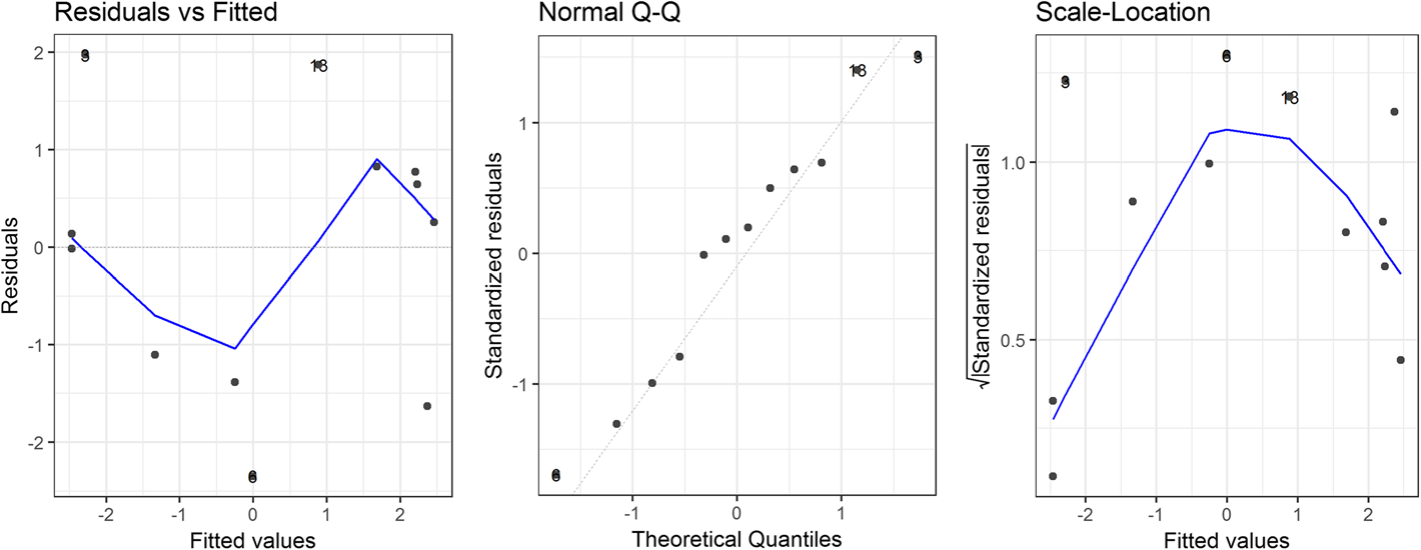
Diagnostic plots for the spring model

**Fig. 4:**
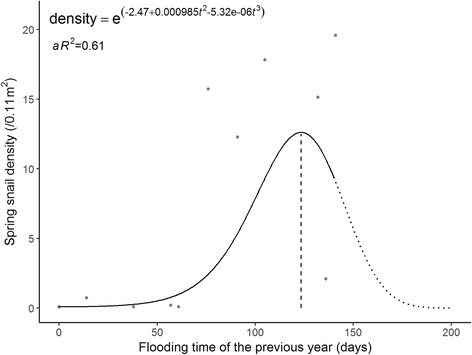
Polynomial regression plot of spring snail density and flooding duration of the previous year

The estimated regression coefficients of the final model for snail density in the spring are shown in Table 1. Both quadratic and cubic terms were significant at the alpha level of 0.10, suggesting a convex relationship between the flooding duration of the previous year and the spring snail density. Diagnostic plots suggested a good fit for the polynomial regression (Fig. 3). Global tests showed that model assumptions of linearity, normality, independence and homoscedasticity were satisfied. The Durbin-Watson test detected no presence of autocorrelation (*P* = 0.35). The spring snail density remained at a low level when the flooding duration of the previous year was fewer than 50 days, was the highest when the flood duration was 123 days, and decreased afterwards (Fig. 4). The model accounted for 61% of the total variation for the spring snail density (*aR*^2^ = 0.61).

**Table 1:** Polynomial regression model for spring snail density over flooding duration of the previous year

Term	Estimate	SE	*P*-value
Intercept	-2.467	0.798	0.013
${t}_2^2$	9.846 × 10^-4^	3.959 × 10^-4^	0.035
${t}_2^3$	-5.317 × 10^-6^	2.787 × 10^-6^	0.089
Model evaluation			
*aR*^2^	0.610		
*F*-statistic	9.594		0.006

*Abbreviation*: *SE* standard error

#### Autumn snail density modeling

**Fig. 5:**
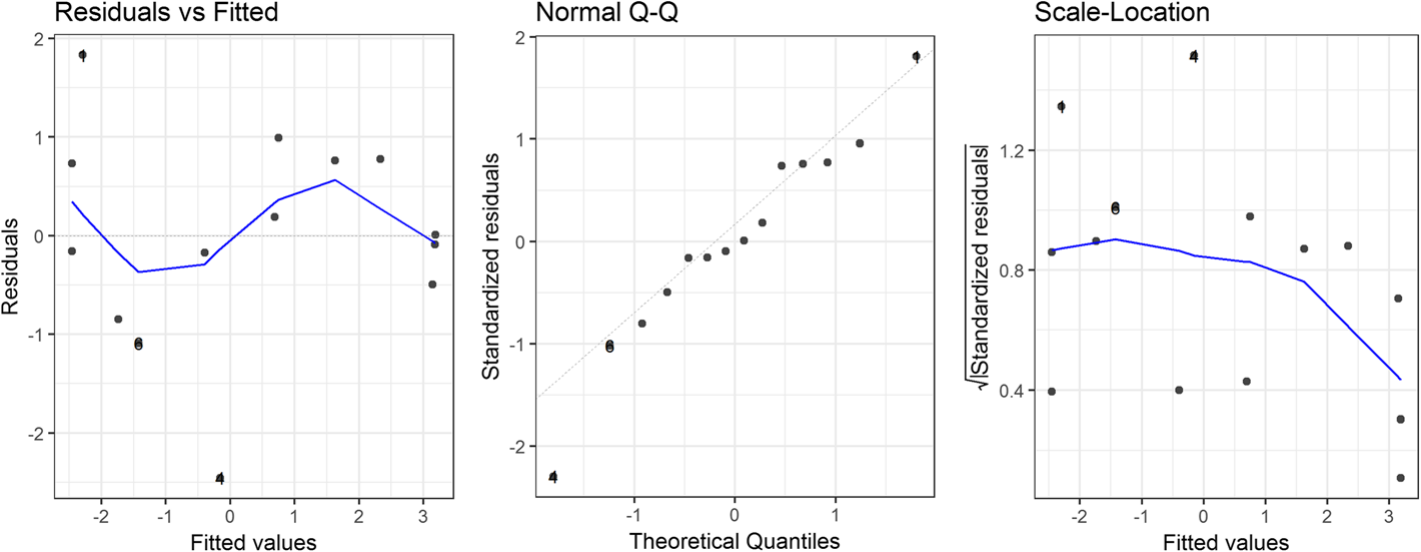
Diagnostic plots for the autumn model

**Fig. 6:**
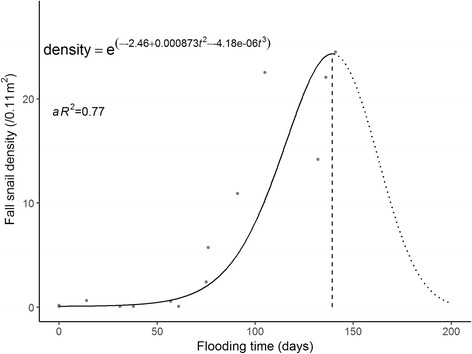
Polynomial regression plot of the autumn snail density and flooding duration of the current year

Table 2 presents the estimated regression coefficients of the final model for the snail density in the autumn. Diagnostic plots (Fig. 5) and global tests showed that model assumptions of linearity, normality, independence and homoscedasticity were satisfied. The Durbin-Watson test detected no sign of autocorrelation (*P* = 0.86). A similar convex relationship was found between the flooding duration of the current year and the snail density (shown in Fig. 6). The autumn snail density remained low when the flooding duration of the current year was fewer than 50 days, increased with flooding days till 139 days, and decreased afterwards. Flooding duration explained 77% of the total variation for the autumn snail density (*aR*^2^ = 0.77).

**Table 2:** Polynomial regression model of autumn snail density over flooding duration of the current year

Term	Estimate	SE	*P*-value
Intercept	-2.456	0.564	0.001
${t}_1^2$	8.733 × 10^-4^	2.809 × 10^-4^	0.010
${t}_1^3$	-4.180 × 10^-6^	1.980 × 10^-6^	0.060
Model evaluation			
*aR* ^2^	0.774		
*F*-statistic	22.260		< 0.001

*Abbreviation*: *SE* standard error

## Discussion

The two study areas are located in Yueyang City, Hunan Province, which is a lake and marshland area and a typical schistosomiasis epidemic region. The warm climate with abundant rainfall, seasonal fluctuations of water level and abundant vegetation provide a suitable environment for the survival and breeding of *O. hupensis* [15]. In addition, the two bottomlands underwent no snail control measures, and therefore were ideal for studying the relationship between flooding duration and snail density.

The spring polynomial regression model showed that the spring snail density remained very low when the number of submerged days was fewer than 50 and then increased with flooding duration up to approximately 123 days and then decreased. Similar results were found for the autumn snail density that the optimal flooding duration was about 139 days. The autumn density remained low when the flooding duration was less than 50 days. The optimal flooding duration derived from the spring model differed from that from the autumn model, which could be caused by overwintering. Our results were similar to previous field observations that in the lake and marshland regions: bottomlands submerged for four to five months each year had the densest distribution of *O. hupensis* snails, followed by those submerged for six to eight months, and those submerged for more than 8 months or less than 1 months had no snails [18]. Submersion is critical for the growth of newly hatched *O. hupensis* snails, but poses negative effects on the survival and spawning of the adult snails [9]. Therefore, the average life expectancy of the snails is about one year [18]. In our study region, the long duration of seasonal flooding each year drowns innumerable adult snails, and surviving snails are the breeding stocks for the coming year’s new generation. A recent study found similar results that the snail density was the highest at about three months of flooding and was at a relatively low level for flooding of more than five months [14]. The difference in optimal flooding duration could be explained by the fact that their study sites included bottomlands inside of the embankment where the submersion was mainly influenced by agricultural irrigation schedules.

Our findings could have some important implications for setting the priority for snail monitoring in the middle and lower reaches of the River Yangtze. Significant changes in hydrology downstream of the dam have been observed following the operation of the Three Gorges Dam in 2003, featuring lower and more stable water levels [7, 8]. The snail density would probably remain low in marshlands with higher elevation and average flooding duration less than two months before the year of 2003. Future monitoring and snail control efforts should focus on marshlands with flooding duration ranging from two to six months. It is noteworthy that those areas with longer historical flooding duration may turn into suitable habitats for snail breeding.

There are some limitations in this study. First, only flooding duration and timing were considered in our study. The two bottomlands in this study were only 15 km apart. No dramatic changes occurred for any of these factors during the study period. Differences in the temperature and rainfall were thought to be negligible between these two bottomlands. Therefore, these factors were not included in the models. Secondly, there was no observation of snail densities with a flooding duration of more than five months. Future studies should cover areas with a longer flooding duration in order to obtain a more complete picture for the quantitative relationship between flooding duration and the snail density. Finally, data on snail densities were collected successively over seven years but autocorrelation was not modelled in our regression analysis; however, Durbin-Watson test did not detect any autocorrelations.

## Conclusions

Flooding duration is an important factor affecting the density of *O. hupensis* snails. There was a convex relationship between flooding duration and the spring or autumn snail density. The snail density was the highest at four to five months of flooding. A too short or too long time of flooding reduced the density of the snail population. Our study provides valuable information for developing evidence-based snail control measures incorporating water level regulations.
